# A Self-Help Program for Memory CD8+ T Cells: Positive Feedback via CD40-CD40L Signaling as a Critical Determinant of Secondary Expansion

**DOI:** 10.1371/journal.pone.0064878

**Published:** 2013-05-23

**Authors:** Jessica A. Shugart, Shelly Bambina, Alejandro F. Alice, Ryan Montler, Keith S. Bahjat

**Affiliations:** Earle A. Chiles Research Institute, Robert W. Franz Cancer Research Center, Providence Cancer Center, Portland, Oregon, United States of America; Federal University of São Paulo, Brazil

## Abstract

The ability of memory CD8+ T cells to rapidly proliferate and acquire cytolytic activity is critical for protective immunity against intracellular pathogens. The signals that control this recall response remain unclear. We show that CD40L production by memory CD8+ T cells themselves is an essential catalyst for secondary expansion when systemic inflammation is limited. Secondary immunization accompanied by high levels of systemic inflammation results in CD8+ T cell secondary expansion independent of CD4+ T cells and CD40-CD40L signaling. Conversely, when the inflammatory response is limited, memory CD8+ T cell secondary expansion requires CD40L-producing cells, and memory CD8+ T cells can provide this signal. These results demonstrate that vaccination regimens differ in their dependence on CD40L-expressing CD8+ T cells for secondary expansion, and propose that CD40L-expression by CD8+ T cells is a fail-safe mechanism that can promote memory CD8+ T cell secondary expansion when inflammation is limited.

## Introduction

A hallmark of the adaptive immune response is that a sublethal infection results in long-lived, antigen-specific immunity [1]. Memory CD8+ T cells (mCD8) are a critical component of the antigen-specific immune response based on their ability to respond rapidly to secondary infection by intracellular pathogens, move into peripheral tissues, and to kill infected cells via recognition of pathogen-derived peptides presented on MHC class I molecules [2], [3]. While both naïve CD8+ T cells and mCD8 receive signal one via TCR-mediated recognition of MHC class I-peptide complexes, these T cell populations differ in their location, phenotype and frequency. Thus, it is not surprising that regulation of a primary versus a secondary CD8+ T cell response involves both common and disparate elements. One such element is CD4+ T cell help. Similar to the primary CD8+ T cell response, the secondary function of mCD8 may require CD4+ T cell help [4], [5], or it may proceed independent of CD4+ T cells [6], [7].

Recent evidence suggests that the secondary function of mCD8 requires professional antigen presenting cells, costimulation and inflammatory cytokines [8]–[11]. Dendritic cell-expressed molecules such as IL-12p70, 4-1BBL and CD86 promote expansion of mCD8 [12]. Expression of these molecules by dendritic cells can occur via multiple signaling pathways, including inflammatory cytokines, TLR ligands, and cell-to-cell interaction. Interaction of T cell-produced CD40L and CD40-expressing dendritic cells (DCs) promotes DC survival, antigen processing and maturation [13], [14]. These findings nonetheless leave many questions to be answered. In what physiologic context is CD40L required? If necessary, are CD4+ T cells solely responsible for delivering CD40L during the recall response? We therefore sought to investigate how the context of a secondary immunization influenced the need for CD4+ T cell help or CD40-CD40L signaling to promote mCD8 secondary expansion.

Expression of CD40L by CD8+ T cells can induce IL-12p70 production by dendritic cells and compensate for the lack of CD4+ T cell help during priming [14], [16], [17]. Here we found that CD4+ T cells were dispensable for mCD8 secondary expansion after immunization, but CD40-CD40L signaling was required. We then showed that a defined subset of memory CD8+ T cells rapidly produce CD40L following recognition of their cognate MHC-peptide complex. The necessity of CD40-CD40L signaling was dictated by the immunization regimen used. When mice were primed with vaccinia virus and boosted with attenuated *L. monocytogenes*, CD40-CD40L signaling was not required for mCD8 secondary expansion. Conversely, when mice were primed and boosted with attenuated *L. monocytogenes*, mCD8 secondary expansion required CD40-CD40L signaling but not CD4+ T cells. The requirement for CD40-CD40L signaling correlated with the in vivo persistence of the boosting vaccine and the elicited inflammatory milieu, but not the dose of antigen. We then showed that in vaccinia virus primed mice, accelerated clearance of the boosting *L. monocytogenes* vaccine by antibiotic treatment limited systemic inflammation and again led to CD40-CD40L-dependent mCD8 secondary expansion. Therefore, the inflammatory context of the secondary vaccination determines the necessity of CD40L expression for expansion, and CD40L-expressing mCD8 represent a uniquely functional T cell subset capable of driving secondary expansion when inflammation is limited.

## Materials and Methods

### Mice

6 to 10 week old C57BL/6 (C57BL/6J), B6.SJL (B6.SJL-*Ptprc^a^ Pepc^b^*/BoyJ), and B6.*Cd40L*-/- (B6.129S2-*Cd40lg^tm1Imx^*/J) mice were obtained from The Jackson Laboratory. All animal protocols were approved by the Earle A. Chiles Research Institute's IACUC.

### Vaccines and immunizations

*L. monocytogenes* strains used for these studies, Δ*actA*-Lm, Δ*actA*-Lm-OVA (Lm-OVA, secreting chicken ovalbumin) and Δ*actA*-Lm-QV (Lm-QV, expressing the class I-restricted vaccinia virus-derived epitopes B8R_20–27_, C4L_125–132_, A42R_88–96_ and K3L_6–15_, in addition to OVA_257–264_) lack the actin-polymerizing protein ActA and are unable to spread from cell to cell [18]–[20]. Bacteria were grown to stationary phase in brain-heart infusion broth, washed in PBS, and injected intravenously in 200 µL total volume. Vaccinia virus WR expressing full-length chicken ovalbumin (VV-OVA) was used for heterologous prime-boost studies. Viral stocks were grown in HeLa cells and frozen. Thawed cell lysates were treated for 30-minutes with 1.25μg/mL trypsin at 37C. Virus was diluted in HBSS and injected intraperitoneally as 1×10^6^ PFU in 200 µL. Mice were immunized intraperitoneally with 5 µg of DEC-205-OVA (generously provided by CellDex Therapeutics) in 200 µL total volume.

**Figure 1:**
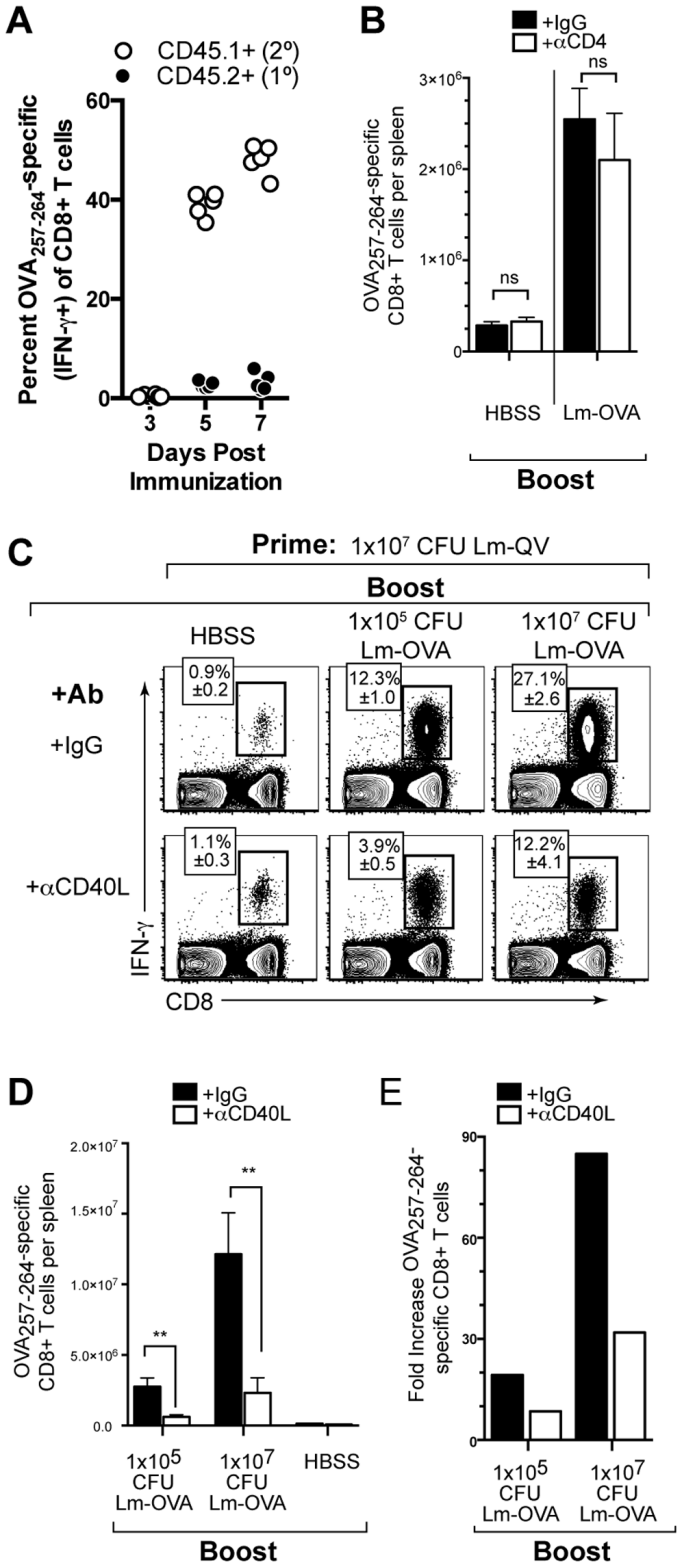
Memory CD8+ T cell expansion following homologous boost is CD4+ T cell independent but CD40L dependent. Mice primed with Lm-OVA were boosted 21 days later with the indicated dose of Lm-OVA. 5 days post-boost, OVA_257–264_-specific CD8+ T cells were enumerated by IFN-γ intracellular cytokine staining. (A) CD8+ splenocytes were transferred from Lm-OVA primed B6.SJL (CD45.1+) mice into C57BL/6 (CD45.2+) recipients. Recipient mice were then treated with Lm-OVA and the frequency OVA_257–264_-specific CD8+ T cells was determined by intracellular IFN-γ staining. (B) Mice were depleted of CD4+ cells at the time of the secondary immunization. Absolute OVA_257–264_-specific CD8+ T cells five days post homologous Lm-OVA prime-boost (mean±SEM, *n* = 5). (C-E) Mice were treated with MR1 (αCD40L) or control antibody during boost. (C) Frequency of OVA_257–264_-specific CD8+ T cells within total CD8+ cells with and without CD40L blockade (mean±SEM, *n* = 5). (D) Absolute number of IFN-γ-producing OVA_257–264_-specific CD8+ T cells (mean±SEM). (E) Fold expansion of OVA_257–264_-specific CD8+ T cells with and without CD40L blockade (mean absolute IFN-γ+ OVA_257–264_ -specific CD8+ T cells per group relative to mean of matched HBSS group). *, P<0.05, **P<0.01, Mann-Whitney. Data are representative of two independent experiments.

We assessed the memory T cell response at >21 days after primary immunization based on the kinetics of contraction of the primary effector CD8+ T cell response elicited by attenuated *L. monocytogenes* and vaccinia virus [21], [22]. Cells were harvested 5 days post-immunization (2 days before the peak of the primary CD8 response in naïve mice) to focus on the expanding mCD8 population. The rationale is demonstrated in Figure 1A, where CD45.1+ cells were transferred from mice primed with Lm-OVA into naïve CD45.2+ recipients. Immunization of the CD45.2+ recipient with Lm-OVA demonstrates the rapid expansion of the antigen-experienced (CD45.1+) CD8+ T cells, and the predominance of those cells over the new CD45.2+ primary response on day 5.

CD4+ T cells were depleted by injecting 300 µg anti-CD4 antibody (clone GK1.5) or rat IgG (polyclonal, BioXCell) three days prior to secondary immunization, followed by 50 µg of antibody one day prior to and two days post-immunization. CD4+ T cell depletion was confirmed by staining splenocytes with anti-CD4 antibody (clone RM4-5) at the time of analysis. The frequency of CD4+ cells was confirmed to be <0.1% for each animal. For CD40L blockade, mice were injected intraperitoneally with 250 µg anti-CD40L (clone MR1) or hamster IgG antibody (BioXCell) three hours prior to immunization, and then again at 24H and 48H post-boost.

### Adoptive transfer of CD8+ T cells

Spleens were harvested from Lm-QV-primed B6.SJL (CD45.1+) mice and CD8+ T cells purified by negative selection (CD8+ T cell enrichment kit, StemCell Technologies). Prior to adoptive transfer, cells were stained with an anti-CD8 antibody to confirm purity of CD8+ T cells (>90%). CD8+ T cells were adoptively transferred into an equal number of primed B6 or CD40L-deficient recipients. The actual number of cells transferred varied per experiment, but was between 5−10×10^6^ CD8+ cells per animal.

### Flow cytometry

Fluorochrome-conjugated antibodies specific for CD45.1 (clone A20), CD45.2 (clone 104), IFN-γ (clone XMG1.2), CD40L (clone MR1) (eBioscience) CD4 (clone GK1.5, except for CD4 depletion experiments when clone RM4-4 was used), CD8α (clone 53-6.7) and TNF (clone MP6-XT22) (BD Bioscience) were used at optimal titers as determined in our laboratory. Peptides for restimulation were obtained from A&A Labs.

Restimulation and staining were performed as previously described [23]. Briefly, splenocytes were restimulated for 5 hours with the indicated peptide (1 µM) in the presence of brefeldin A (GolgiPlug, BD Biosciences). All peptides were reconstituted in DMSO. Unstimulated controls (DMSO only) were used to assess nonspecific protein production for each animal. Cells were stained for surface antigens, and then fixed (Cytofix/Cytoperm buffer, BD Bioscience) and stored at −80C (in Cytofix/Cytoperm buffer) until further analysis. For intracellular cytokine staining, frozen cells were thawed, permeabilized (Perm Wash buffer, BD Biosciences), and stained for intracellular IFN-γ, TNF, and CD40L. Samples were acquired on an LSRII flow cytometer (BD Biosciences) and analyzed using FlowJo (Treestar software) and SPICE v5.3 (http://exon.niaid.nih.gov/spice/).

### Bacterial clearance

Spleens and livers were harvested into buffer containing 0.2% Igepal at the indicated times following boost with 1×10^7^ cfu Δ*actA-Lm*-QV. Organs were homogenized using an Omni Prep multi-sample homogenizer (Omni International) and plated on BHI plates containing streptomycin. Colonies were enumerated and CFU/organ was calculated.

### Serum cytokine analysis

Serum cytokines were determined using the Mouse Inflammation Cytometric Bead Array (BD Biosciences). Samples were acquired on an LSRII flow cytometer and the exported data were analyzed using the CBA Analysis Plugin for Excel.

### Statistical Analyses

Data represent at least two independent experiments, each containing 5 mice per experimental group. Error bars represent the standard error of the mean (SEM). Statistical analyses were performed for each independent experiment using a one-way ANOVA with Newman-Keuls post-test, or Mann-Whitney test where indicated.

## Results

### Memory CD8+ T cell expansion following homologous prime-boost is CD4+ T cell independent but CD40L dependent

We first asked if mCD8 secondary expansion following homologous prime-boost required CD4+ T cell help. Evaluating the antigen-specific response 5 days post-boost minimizes the contribution of newly primed CD8+ T cells to the analysis (Figure 1A). In all experiments, mice were fully immunocompetent at the time of the initial vaccination. Blocking or depleting antibodies were only used during the secondary immunization. Finally, to avoid the complications of neutralizing vector-specific antibodies, we used a live-attenuated *L. monocytogenes*-based vaccine for homologous prime-boost [24].

C57BL/6 (B6) mice were vaccinated with an attenuated *L. monocytogenes* expressing ovalbumin (Lm-OVA). Three weeks later, mice were depleted of CD4+ cells and boosted with Lm-OVA. Five days after the boosting immunization, OVA_257–264_-specific CD8+ T cells were enumerated using intracellular cytokine staining (Figures 1B,C). Neither the expansion nor the functionality of the memory CD8+ T cell population was affected by depletion of CD4+ T cells prior to boost. Therefore, homologous prime-boost with a live-attenuated *L. monocytogenes*-based vaccine elicited CD8+ T cell secondary expansion independent of CD4+ T cell help.

Given that CD4+ T cells were not required for mCD8 secondary expansion, we hypothesized that CD40L was also dispensable. To test this, B6 mice vaccinated with Lm-QV were boosted 21 days later with Lm-QV in the presence or absence of CD40L blockade. Interestingly, secondary expansion was limited by the absence of CD40-CD40L signaling (Figures 1D-E). Increasing the dose of Lm-QV used for the boost vaccination did not overcome the need for CD40–CD40L signaling. Therefore, optimal secondary mCD8 expansion following homologous prime-boost vaccination is independent of CD4+ T cell help, but is substantially enhanced by CD40–CD40L signaling.

### Heterologous prime-boost maximizes mCD8 secondary expansion independent of CD40L signaling

**Figure 2:**
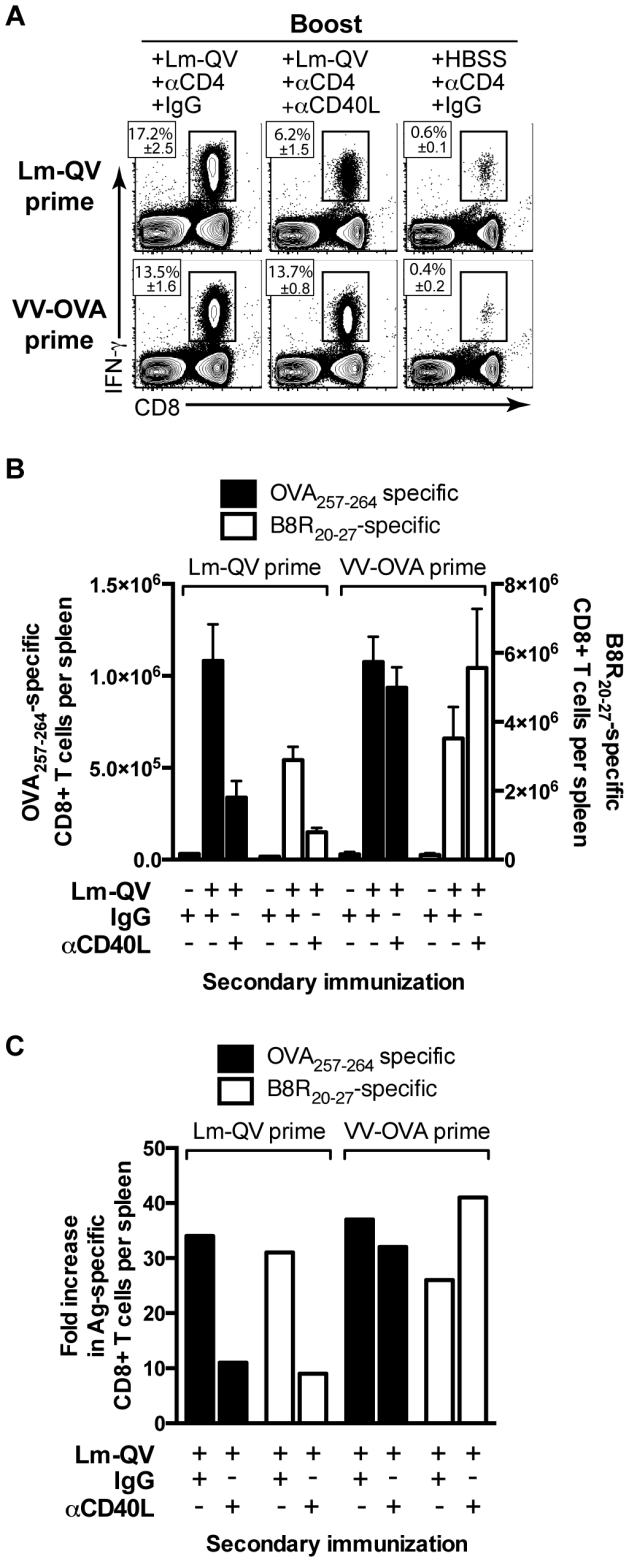
CD40L is required for mCD8+ T cell expansion following homologous (but not heterologous) boost, in the absence of CD4+ T cells. Mice were primed with 10^7^ cfu Lm-QV or 10^6^ pfu VV-OVA and boosted 21 days later with 10^5^ cfu Lm-QV. Starting on day 20 (1 day pre-boost), mice were depleted of CD4+ T cells. MR1 (αCD40L) or control antibody was administered to indicated groups during boost. OVA_257–264_-specific CD8+ T cell responses were assessed 5 days post-boost by intracellular cytokine staining. (A) Frequency of OVA_257–264_-specific CD8+ T cells within total CD8+ cells (mean±SEM, *n* = 5). (B) Total IFN-γ+ OVA_257–264_-specific or B8R_20–27_-specific mCD8+ T cells per spleen (mean±SEM). (C) Fold expansion of OVA_257–264_-specific or B8R_20–27_-specific CD8+ T cells after homologous or heterologous boost. (mean absolute IFN-γ+ OVA_257–264_ -specific CD8+ T cells per group relative to mean of matched HBSS group). Data are representative of two independent experiments.

The use of unique vectors expressing a common target antigen (i.e. heterologous prime-boost) can significantly improve the magnitude and potency of the T cell response relative to repeated vaccination with the same vector [25], [26]. We asked if mCD8 secondary expansion after a heterologous prime-boost was also dependent on CD40–CD40L signaling. To address this question, B6 mice were primed with either vaccinia virus expressing ovalbumin (VV-OVA) or Lm-QV. Three weeks later, mice were depleted of CD4+ cells and then boosted with Lm-QV, with or without CD40L blockade. Five days later the expansion of antigen-specific CD8+ T cells was measured (Figure 2A). In contrast to homologous prime-boost, mCD8 secondary expansion after heterologous prime-boost (VV-OVA prime, Lm-QV boost) was not dependent on CD40–CD40L signaling (Figures 2B–D). Similar patterns of expansion were observed for B8R_20–27_, and OVA_257–264_, CD8+ T cells. Because CD4+ cells were depleted prior to the boost, these cells could not be the source of CD40L. Thus, altering the priming and boosting vaccine vectors promoted optimal mCD8 secondary expansion without the need for CD40–CD40L signaling.

### A distinct subset of memory CD8+ T cells produces CD40L

**Figure 3:**
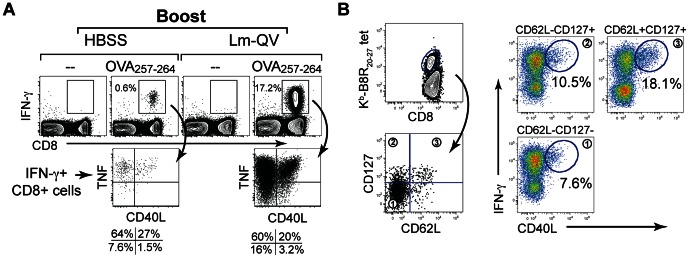
CD40L expressing mCD8+ T cells promote secondary expansion following homologous boost. (A) Mice primed with 10^7^ cfu Lm-QV were boosted 21 days later with HBSS or 10^5^ cfu Lm-QV. 5 days following boost, splenocytes were restimulated with OVA_257–264_ in the presence of brefeldin A, and then stained with antibodies for CD4, CD8 (surface), IFN-γ,TNF and CD40L (intracellular). Left panels demonstrate CD40L expression in non-boosted, day 21+5 OVA_257–264_-specific CD8+ T cells. Right panels represent OVA_257–264_-specific CD8+IFN-γ+TNF+CD40L+ cells 5 days after boosting with Lm-QV. (B) Mice were primed and boosted with 10^7^ CFU Lm-QV. Five days after the boost, CD8+B8R_20–27_-specific splenocytes were sorted into central memory (CD127+CD62L+), effector memory (CD127+CD62L-) and effector (CD127-CD62L-) subsets. Sort purified cells were incubated for five hours with B8R_20–27_ peptide in the presence of brefeldin A, and then stained for CD4, CD8, and intracellular IFN-γ and CD40L. Data are representative of two independent experiments.

The divergent roles of CD4+ T cell help and CD40L signaling during homologous boost identified a role for non-CD4+ T cell expression of CD40L during secondary expansion. We asked if memory CD8+ T cells produce CD40L, and if so, if CD40L expression was homogeneous amongst the CD8+ T cells or restricted to subset of these cells. Lm-QV-primed mice were boosted with Lm-QV or HBSS 21 days later, and memory CD8+ T cells were analyzed for expression of CD40L 5 days following boost. While CD40L was undetectable in unstimulated CD8+ T cells, a brief (5 hour) restimulation with peptide and brefeldin A revealed a discrete subset of CD40L-expressing CD8+ T cells (Figure 3A). Importantly, CD40L was only detectable when cells were restimulated in the presence of the transport inhibitor brefeldin A. Using this technique, CD40L is retained within the cell and can be detected by intracellular staining. Co-staining with antibodies to IFN-γ, TNF, and CD40L revealed that not all IFN-γ and TNF double-positive CD8+ T cells can produce CD40L, but all CD40L-producing CD8+ T cells do produce IFN-γ and TNF. The subset of CD40L-producing CD8+ T cells was detectable after one or two immunizations, and was maintained within the memory CD8+ T cell pool, suggesting that a subset of mCD8 are programmed to express CD40L.

To identify the CD8+ T cell subsets capable of CD40L expression, we sorted antigen-specific T cells (identified by staining with K^b^-B8R_20–27_ tetramers) into central memory (CD62L+CD127+), effector memory (CD62L-CD127+) and effector (CD62L-CD127−) subsets [27]. These populations were subsequently stimulated for 5 hours with the B8R_20–27_ peptide in the presence of brefeldin A and then stained for intracellular IFN-γ and CD40L (Figure 3B). CD8+ T cells with a memory phenotype (CD127+) were more likely to produce CD40L than the CD127- effector population. Within those CD127+ memory cells, the CD62L+ central memory subset was the most likely to produce CD40L. Based on these results, we hypothesize that CD62L+CD127+ central memory CD8+ T cells are the most capable of producing CD40L.

**Figure 4:**
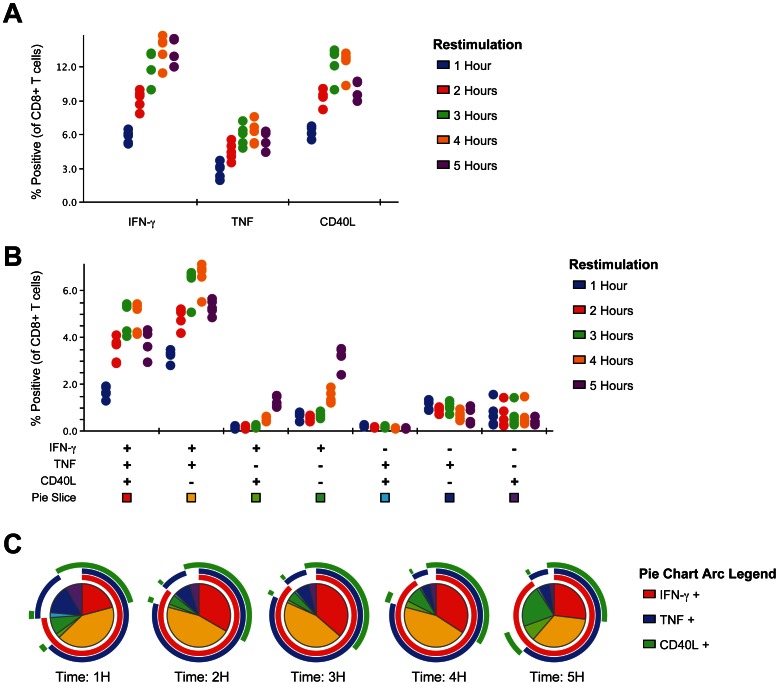
Rapid expression of CD40L by CD8+ T cells following restimulation. Mice were primed with 1×10^7^ cfu Lm-QV and spleens harvest 7 days later. Splenocytes were restimulated in vitro with B8R_20–27_ for the indicated time and then stained for intracellular IFN-γ, TNF and CD40L. (A) Univariate analysis of intracellular IFN-γ, TNF or CD40L within CD8+ T cells after restimulation. (B) Multivariate analysis of IFN-γ, TNF and CD40L over time within CD8+ T cells. (C) Protein expression profile of B8R_20–27_ –specific CD8+ T cells after restimulation. Pie slices correspond to color legend in panel B. Outer arcs indicate slices expressing the phenotype of the inner slices. Each point in panels A and B indicates a single animal (5 mice per group), panel C represents the median of data from panel B.

To better understand the kinetics of CD40L expression by CD8+ T cells, splenocytes from mice primed with Lm-QV were restimulated for 1–5 hours with B8R_20–27_ and then stained for intracellular IFN-γ, TNF and CD40L (Figure 4). All three proteins were rapidly produced by CD8+ T cells, visible after 1 hour of restimulation (Figure 4A). The frequency of triple-positive CD8+ T cells increased over the first 4 hours of restimulation. After 5 hours of restimulation, expression of TNF and CD40L begins to wane and more cells producing only IFN-γ are seen (Figure 4B, C).

### CD40L-expressing memory CD8+ T cells are sufficient to amplify secondary expansion

**Figure 5:**
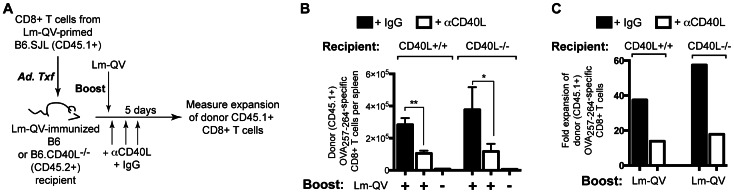
CD40L expressing mCD8+ T cells promote secondary expansion following homologous boost. (A) Experimental design: Donor (B6.SJL) mice and recipient (C57BL/6 and CD40L-deficient) mice were concurrently primed with 10^7^ cfu Lm-QV. 21 days later, CD8+ T cells were purified from B6.SJL spleens and transferred into B6 and B6.*Cd40L*-/- mice. 24 h post-transfer, recipients were boosted with 5×10^6^ cfu Lm-QV in the presence of αCD40L or control antibody. OVA_257–264_-specific memory CD8+ T cell responses were assessed 5 days following boost using intracellular cytokine staining for IFN-γ (*n* = 5). (B) Total OVA_257–264_-specific (IFN-γ+) CD45.1+CD8+ T cells per animal (mean, ±SEM). *, P<0.05, **, P<0.01, Mann-Whitney. (C) Fold expansion of donor CD45.1+ IFN-γ + OVA_257–264_-specific CD8+ T cells. Data are representative of three independent experiments.

We next asked if mCD8-specific CD40L was indeed sufficient to maximize mCD8 secondary expansion. To test this, we primed wild-type CD8+ T cells (CD8+*Cd40L*+/+) by immunizing B6.SJL mice with Lm-QV. Three weeks later, we transferred these antigen-experienced CD8+ T cells into recipients lacking CD40L (B6.*Cd40L*-/-) (Figure 5A). In this system, the only cells capable of producing CD40L are the transferred CD8+*Cd40L*+/+ T cells. Recipient mice were primed prior to adoptive transfer to establish Lm-specific immunity and recreate the conditions of homologous prime-boost. Preliminary experiments confirmed that Lm-QV was cleared with similar kinetics from previously immunized B6 and B6.*Cd40L*-/- mice (data not shown). 24 hours after adoptive transfer, mice were immunized with Lm-QV and 5 days later, Ag-specific CD8+ T cells were enumerated. Donor CD8+*Cd40L*+/+ cells expanded similarly in B6 or B6.*Cd40L*-/- recipients (Figures 5B,C), and CD40L blockade impaired secondary expansion in both scenarios. Thus, when only mCD8 can express CD40L, the secondary expansion of those mCD8 remains intact via a CD40L-dependent mechanism.

### Rapid clearance of homologous vaccine vector during secondary immunization limits inflammation and correlates with increased CD40L dependence

**Figure 6:**
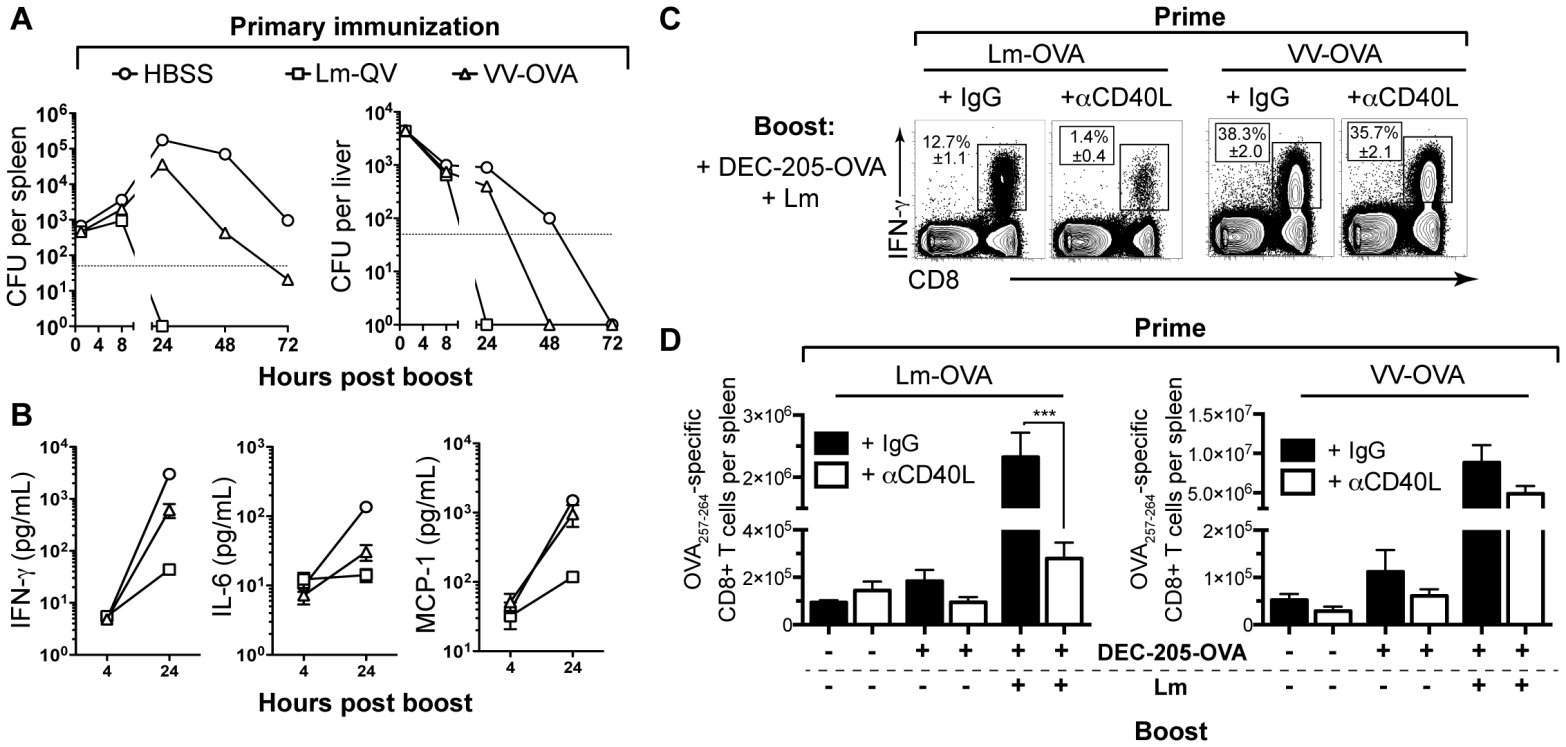
Accelerated clearance of homologous vaccine vector limits inflammation and correlates with increased CD40L dependence. (A–B) Mice were primed with 10^7^ cfu Lm-OVA, 10^6^ pfu VV-OVA, or HBSS and boosted 21 days later with 10^7^ cfu Lm-QV (*n* = 5). (A) Spleens and livers were harvested at 24, 48, and 72 hours following boost, and cfu were enumerated by plating organ homogenates (mean, ±SEM). (B) Serum cytokine levels at 4 and 24 h following boost (mean, ±SEM). (C–E) Mice were primed with 10^7^ cfu Lm-OVA or 10^6^ pfu VV-OVA and boosted 21 days later with 5 µg DEC205-OVA alone, 5 µg DEC205-OVA with 10^5^ cfu Lm-OVA, or HBSS. αCD40L or control antibody was administered to the indicated groups during boost. OVA_257–264_-specific memory CD8+ T cell responses were assessed 5 days post-boost using intracellular cytokine staining for IFN-γ (*n* = 5). (C) Representative plots of OVA_257–264_-specific T cell enumeration (median shown, ±SEM). (D) Total OVA_257–264_-specific (IFN-γ+) CD8+ T cells per animal (mean, ±SEM). *, P<0.05, **, P<0.01, ***, P<0.001, ANOVA. Data are representative of two independent experiments.

Antigen-specific mCD8 are the principal mediators of protective immunity to *L. monocytogenes* [28]. We hypothesized that the discordant requirement for CD40-CD40L signaling between homologous and heterologous prime-boost would be explained by different rates of vaccine clearance following secondary immunization. Although Lm-QV and VV-OVA share 5 class I-restricted epitopes, the adaptive response toward the other 3,000+ proteins expressed by Lm-QV following a homologous boost could play a significant role in vaccine clearance. To determine how the homologous or heterologous prime affected clearance of the secondary vaccine, B6 mice were primed with VV-OVA or Lm-QV and then three weeks later, boosted with Lm-QV. We enumerated Lm-QV CFU in the spleen and liver between 1 and 72 hours after vaccination (Figure 6A), and quantified serum cytokines at 4 and 24 hours (Figure 6B). As predicted, vaccine was cleared substantially faster in vaccinated versus naïve mice. Mice primed with Lm-QV completely cleared the Lm-QV boost within 24 hours of vaccination. Mice primed with VV-OVA required 72 hours before Lm-QV was eradicated from both spleen and liver.

Delayed clearance of the boosting vector increased the innate inflammatory response, where serum concentrations of IFN-γ, MCP-1 and IL-6 negatively correlated with the rate of Lm-QV clearance (Figure 6B). Therefore, the breadth of vector specific immunity correlates with the kinetics of vaccine clearance during secondary immunization, the innate inflammatory response, and the dependence on CD40L to promote mCD8 secondary expansion.

In addition to limiting inflammation, accelerated clearance of Lm-QV may decrease the quantity of antigen available for processing and presentation. If antigen load were the determinate of CD40L-dependence or independence, then normalizing the antigen concentration should eliminate the disparity in CD40L-dependence between homologous and heterologous prime-boost. To test this, B6 mice were primed with Lm-OVA or VV-OVA. Three weeks later, mice were boosted using DEC-205-OVA. This DEC-205-specific antibody delivers ovalbumin to DEC-205-expressing dendritic cells for processing and presentation [29]. We then injected a *L. monocytogenes* strain with the same attenuating deletion as Lm-OVA, but without the OVA antigen expression cassette. In this model system, all mice receive an equivalent dose of antigen (ovalbumin, delivered by DEC-205-OVA) irrespective of how quickly the Lm is cleared. The Lm serves only as an adjuvant in these experiments. Finally, mice were treated with anti-CD40L or hamster IgG and the frequency of OVA_257–264_-specific CD8+ T cells was determined five days later (Figure 6C). Immunization with DEC-205-OVA alone does not elicit mCD8 secondary expansion, reinforcing the need for some degree of inflammation for secondary expansion. Consistent with our hypothesis, mCD8 in mice primed with Lm-OVA underwent secondary expansion that was CD40L dependent (Figure 6D). Conversely, mice primed with VV-OVA demonstrated mCD8 secondary expansion that was independent of CD40-CD40L signaling. Thus, independent of antigen concentration, vector-specific immunity determines the necessity of CD40-CD40L signaling for mCD8 secondary expansion.

### Truncating secondary infection recapitulates the CD40-CD40L dependence of the homologous boost

**Figure 7:**
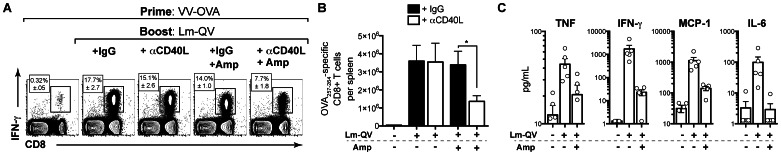
Antibiotic treatment following heterologous boost recapitulates dependence on CD40L for mCD8+ T cell expansion. Mice were immunized with 10^6^ pfu VV-OVA and boosted 21 days later with 5×10^6^ cfu Lm-QV in the presence of MR1 or control antibody treatment. 8 hours following boost, indicated groups were treated with ampicillin to accelerate bacterial clearance. OVA_257–264_-specific memory CD8+ T cell responses were assessed 5 days post-boost using intracellular cytokine staining for IFN-γ (*n* = 5). (A) Representative intracellular cytokine staining (median shown, ±SEM). (B) Total OVA_257–264_-specific (IFN-γ+) CD8+ T cells (mean, ±SEM). *, P<0.05, Mann-Whitney. (C) Serum cytokines 24 h post-boost in untreated versus ampicillin-treated animals (mean, ±SEM). Data are representative of three independent experiments.

If delayed clearance of Lm allows CD40-CD40L independent mCD8 secondary expansion after a heterologous boost, then accelerating Lm clearance should increase the dependence on CD40-CD40L signaling. To test this hypothesis, mice were primed with VV-OVA and then three weeks later, boosted with Lm-QV with and without CD40L blockade. Mice were treated with ampicillin eight hours after secondary immunization to accelerate Lm-QV clearance. Five days after boosting, mCD8 secondary expansion was assessed by enumerating antigen-specific CD8+ T cells. Accelerating Lm-QV clearance with ampicillin treatment recapitulated the CD40-CD40L dependence of mCD8 secondary expansion observed after homologous prime-boost (Figures 7A,B). CD40L dependence correlated with decreased concentrations of IFN-γ, IL-6, MCP-1 and TNF in the serum (Figure 7C). These results confirm that accelerated clearance of the boosting vaccine vector decreases inflammation thereby necessitating CD40L expression by mCD8 to maximize secondary expansion.

## Discussion

Our data demonstrate that CD40L-expressing mCD8 can promote their own secondary expansion when inflammation is limited. It is appreciated that antigen-specific CD8+ T cells can compensate for a lack of CD4+ T cell help during priming via a CD40L-dependent mechanism [15], [16]. In addition to MHC-peptide and costimulatory signals, CD8+ T cell priming requires a third signal, which can be delivered via IL-12p70 or type I IFN [30]. DCs express CD40, and combined signals via TLRs and CD40 result in production of IL-12p70 and upregulation of costimulatory molecules such as CD70, CD86 and 4-1BBL [12], [17]. In vitro, CD8+ T cells promote CD40-dependent IL-12p75 production by CD8α+ DCs [31]. In addition, the IFN-γ-driven production of IL-18 by the DC can be important for secondary expansion and may be impacted by CD40L-expressing CD8+ T cells [32]. While the minimal ‘signal three’ requirements for the antigen-experienced CD8+ T cell are unclear, we hypothesize that rapid production of CD40L, together with IFN-γ, licenses the antigen-presenting DC for IL-12p70 and IL-18 production, which subsequently drives secondary expansion. This process is likely to occur through the CD8α+ DC, as they are uniquely equipped for cross-presentation of exogenous antigen via the class I pathway [33].

In the context of CD8+ T cell-mediated immunity to intracellular microbes, the capacity for mCD8 to proliferate in response to low levels of inflammation is critical to their functionality. The initial infectious dose during a typical infection is unlikely to elicit the kind of immediate cytokine release observed in our vaccination model. One could envision that during the course of a natural secondary infection, a limited amount of inflammation will accompany presentation of pathogen-derived peptides on class I MHC. By virtue of their location, increased frequency, and increased ability to respond to peptide-MHC, the antigen-specific mCD8 population can rapidly respond to prevent disseminated infection. In this scenario, it could be the intrinsic CD40L-expression by mCD8 that synergizes with the localized inflammatory response and drives mCD8 secondary expansion. Thus, CD40L-expression by antigen-specific CD8+ T cells may reflect the potency of those cells and predict their effectiveness in preventing infection.

These studies offer insight into the favorable adaptive immune responses elicited by heterologous prime–boost immunization regimens [34], [35]. The primary hurdle this approach overcomes is the induction of vector-specific humoral immunity and attenuated responsiveness to subsequent vaccinations. In the case of viral-based vaccine vectors, antibodies specific for viral proteins impair viral entry, limit expression of the encoded disease-specific antigen and ultimately, presentation of the relevant MHC-peptide complexes [36]. Yet despite the demonstrated potency of this vaccination approach, little is known mechanistically beyond the avoidance of neutralizing humoral immunity [37]. Our data demonstrate that secondary immunization with a heterologous vaccine vector reduces the speed with which the secondary vaccine is cleared, increasing the innate inflammatory response and overcoming not only the requirement for CD4+ T cell help, but also CD40-CD40L signaling.

While these studies address previous discrepancies regarding the necessity of CD4 help and CD40 signaling during the secondary response, they also pose a new set of questions. Why do only a subset of antigen-specific CD8+ T cells express CD40L after restimulation? Were these cells primed differently, or do they express TCRs with higher affinity for their cognate peptide-MHC? While the conditions required during priming to elicit a CD40L-expressing mCD8 population are unknown, it is unlikely that all vaccines are equivalent in this respect - especially if the goal is to activate a self-reactive CD8+ T cell population. In this case, the use of a heterologous boosting vector that elicits a substantial inflammatory response will promote secondary expansion of mCD8 whether or not those mCD8 express CD40L. So while the expression of CD40L by CD8+ T cells may reflect the priming conditions, the necessity of those CD40L-expressing mCD8 for a recall response can be mitigated through the use of a pro-inflammatory heterologous vaccine.

The utility of heterologous prime-boost should also be considered in light of pre-existing immunity to many microbial vaccine vectors. For example, clinical trials using *L. monocytogenes*-based vaccine vectors must contend with patients that have been exposed to *L. monocytogenes* repeatedly over their lifetime [24], [38], [39]. In this context, the presence of *Listeria*-specific CD8+ T cells could either help (via CD40L-dependent licensing of DCs) or hurt the T cell response (via rapid killing of APCs presenting both *Listeria*- and tumor-associated epitopes). Retrospective analyses of banked PBMC from human vaccination clinical trials may be informative in this regard, based on the hypothesis that vaccines that elicit CD40L-expressing CD8+ T cells would provide superior antigen-specific immunity.
